# One‐Step Synthesis of White‐Light‐Emitting Carbon Dots for White LEDs with a High Color Rendering Index of 97

**DOI:** 10.1002/advs.202206386

**Published:** 2023-02-23

**Authors:** Zishan Yan, Tong Chen, Lingpeng Yan, Xinghua Liu, Jingxia Zheng, Fu‐de Ren, Yongzhen Yang, Bin Liu, Xuguang Liu, Bingshe Xu

**Affiliations:** ^1^ Key Laboratory of Interface Science and Engineering in Advanced Materials Ministry of Education Taiyuan University of Technology 030024 Taiyuan P. R. China; ^2^ Shanxi‐Zheda Institute of Advanced Materials and Chemical Engineering 030032 Taiyuan P. R. China; ^3^ College of Materials Science and Engineering Taiyuan University of Technology 030024 Taiyuan P. R. China; ^4^ School of Energy and Power Engineering North University of China 030051 Taiyuan P. R. China; ^5^ School of Chemical Engineering and Technology North University of China 030051 Taiyuan P. R. China

**Keywords:** color rendering index, multicolor, white light‐emitting diodes, white‐light‐emitting carbon dots

## Abstract

White‐light‐emitting carbon dots (WCDs) show innate advantages as phosphors in white light‐emitting diodes (WLEDs). For WLEDs, the color rendering index (CRI) is the most important metric to evaluate its performance. Herein, WCDs are prepared by a facile one‐step solvothermal reaction of trimellitic acid and *o*‐phenylenediamine. It consists of four CDs identified by column chromatography as blue, green, yellow, red, and thus white light is a superposition of these four types of light. The mixture of the four CDs undergoes Förster resonance energy transfer to induce the generation of white light. The photoluminescence of WCDs originates from the synergistic effect of carbon core and surface states. Thereinto, the carbon core states dominate in RCDs, and the increase of amide contents and degree of conjugation promote the redshift of the emission spectra, which is further confirmed by theoretical calculations. In addition, a high CRI of 97 is achieved when the WCDs are used as phosphors to fabricate WLEDs, which is almost the highest value up to now. The multicolor LEDs can also be fabricated by using the four multicolor CDs as phosphors, respectively. This work provides a novel approach to explore the rapid preparation of low‐cost, high‐performance WCDs and CDs‐based WLEDs.

## Graphical abstract

The white‐light‐emitting carbon dots are synthesized by a one‐step solvothermal method of trimellitic acid and *o*‐phenylenediamine, which consists of four types of carbon dots emitting blue, green, yellow, and red light. Five kinds of light‐emitting diodes can be prepared using the above carbon dots, among which the color rendering index of white light‐emitting diode is as high as 97.

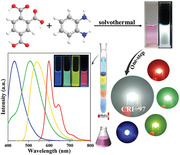

## 1 Introduction

White light‐emitting diodes (WLEDs) are the most prospective high‐tech fields of the 21^st^ century owing to their compact volume, simple structure, and long operational life, and are almost suitable for all indoor and outdoor lighting.^[^ ^1^ ^]^ Currently, they have been widely used in lighting and display,^[^ ^2^ ^]^ and show significant application potential in visible light communication technology.^[^ ^3^ ^]^ There are three main strategies to realize the white light emission of phosphor‐converted LEDs, namely, blue LED chips coupled with yellow emissive phosphor,^[^ ^4^ ^]^ Ultraviolet (UV) chips coupled with red, green, and blue (RGB) emissive phosphors,^[^ ^5^ ^]^ and UV chips coupled with white emissive phosphor.^[^ ^6^ ^]^ For the first strategy, the color rendering index (CRI) of WLEDs is relatively lower than the latter two strategies owing to the lack of long‐wavelength light. CRI reflects the ability of the light source to present the true color of the object. A low CRI affects the recognition of the color of the object by the human eye, which can easily cause visual fatigue and lead to myopia. Meanwhile, the lighting devices for some places, such as museums and studios, require precise color contrast, the CRI needs to be over 90. Although the WLEDs made of RGB phosphors combined with UV chips exhibit high CRIs, they are not suitable for large‐scale preparation and application owing to the difficulty in color balance between phosphors, complicated fabrication, and high cost. White emissive phosphor can make up for the above deficiencies and has the advantages of low manufacturing cost and facile synthesis, making it an ideal candidate for WLEDs.^[^ ^7^ ^]^ In this case, the preparation of white emissive materials becomes a research hotspot.^[^ ^8^ ^]^

Carbon dots (CDs), as an emerging luminescent material, show great advantages and application prospects in the field of WLEDs by virtue of their low toxicity,^[^ ^9^ ^]^ tunable fluorescence emission,^[^ ^10^ ^]^ low cost,^[^ ^11^ ^]^ and excellent photostability.^[^ ^12^ ^]^ They can achieve panchromatic fluorescence emission in the visible light region,^[^ ^13^ ^]^ even white light emission.^[^ ^14^ ^]^ Although a large number of CDs have been used in WLEDs,^[^ ^15^ ^]^ most WLEDs are usually achieved by mixing a variety of CDs with different preparation paths or by mixing CDs with rare earth‐based phosphors,^[^ ^16^ ^]^ leading to a series of problems such as complicated preparation, self‐absorption losses, and poor reproducibility. Currently, one‐step synthesis of white‐light‐emitting CDs (WCDs) has been reported by some research groups.^[^ ^14^, ^17^ ^]^ Unfortunately, most of the WCDs undergo complex purification methods or the narrow emission spectra of WCDs result in the low CRI of WLEDs.^[^ ^18^ ^]^ Therefore, it is of consequence to propose an effective strategy for the facile one‐step synthesis of WCDs and apply the WCDs to realize WLEDs with high CRI.

In this work, a simple and rapid strategy for the preparation of WCDs is established by a one‐step solvothermal method using trimellitic acid (TMLA) and *o*‐phenylenediamine (*o*‐PD) as raw materials. The photoluminescent (PL) spectra and column chromatography show that the synthesized WCDs are composed of four kinds of CDs, namely BCDs, GCDs, YCDs, and RCDs. Subsequently, a series of experimental results prove that the main factor of the PL redshift of CDs is the synergistic effect of the carbon core and surface states, and there is Förster resonance energy transfer (FRET) between multicolor CDs. Theoretical calculations of simplified models of CDs further confirm that the redshift of CDs originates from the large conjugation domain and high amide content, which is highly consistent with the experiment. Finally, CDs were applied to the fabrication of multicolor and white LEDs, in which a WLED was obtained from WCDs, with Commission International de l'Eclairage (CIE) coordinates of (0.32, 0.35), correlated color temperature (CCT) of 6009 K and CRI as high as 97.

## 2 Results and Discussion

**Scheme 1:**
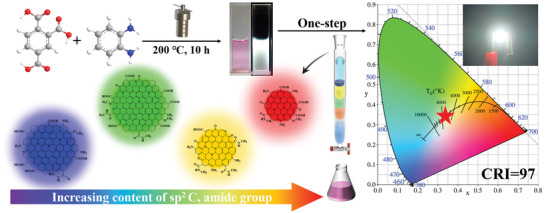
Schematic illustration of the preparation procedure of WCDs and WLEDs.

**Figure 1:**
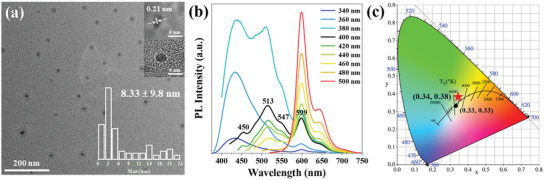
a) TEM image of WCDs. Insets are HRTEM images (upper right) and size distribution histograms (lower right). b) PL spectra of WCDs at different *λ*_ex_. c) CIE coordinates of WCDs in ethanol solution (*λ*_ex_ = 400 nm, 0.625 mg mL^−1^).

As shown in **Scheme** **1**, the WCDs were obtained by a one‐step solvothermal method using TMLA and *o*‐PD as precursors. The reaction temperature, time, and ratio of TMLA and *o*‐PD are 200 °C, 10 h, and 1:2, respectively. The detailed process of preparation and modulating parameters is provided in the Experimental Section and Figure S1, Supporting Information. Interestingly, the as‐prepared WCDs were strong hydrophobic, thus pure solid WCD powders were quickly obtained by precipitation in water, which can remove the impurities without any additional purification steps. This simple purification step can facilitate the large‐scale preparation of pure solid WCDs. The WCDs were dispersed in ethanol to form a clear pink solution in daylight and emit bright white fluorescence under a 400 nm lamp (Scheme 1). The transmission electron microscopy (TEM) image reveals that the WCDs are well‐dispersed with a wide particle size distribution (1–24 nm), and an average particle size of 8.33 nm (**Figure** **1**a). The high‐resolution TEM (HRTEM) images show that some of the WCDs exhibit well‐resolved lattice fringes with a spacing of 0.21 nm, corresponding to the (100) lattice spacing of graphene,^[^ ^19^ ^]^ and some without obvious lattice fringes are also present (inset in Figure 1a). It indicates that the WCDs may have undergone different dehydration and carbonization processes, resulting in a variety of CDs with different structures. The PL spectra of WCDs show four obvious emission peaks at 450, 513, 547, and 599 nm when the excitation wavelength (*λ*_ex_) is 400 nm (Figure 1b), covering the whole visible region (400–750 nm). When the concentration of WCDs in ethanol solution is 0.625 mg mL^−1^, the CIE coordinates are (0.34, 0.38), which is close to (0.33, 0.33) of pure white light (Figure 1c). The multiple emission peaks probably originate from different color‐emitting CDs based on the TEM results. Meanwhile, the excitation‐independent property and the relative peak intensity as a function of *λ*_ex_ indicate that the WCDs are composed of different fluorescence emission centers.

**Figure 2:**
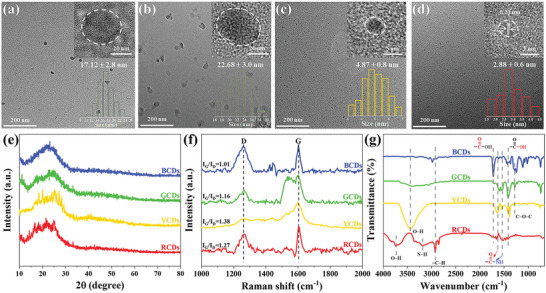
a–d) TEM images of a) BCDs, b) GCDs, c) YCDs, and d) RCDs. Insets are the corresponding HRTEM images (upper right) and size distribution histograms (lower right). e) XRD patterns, f) Raman spectra, and g) FTIR spectra of BCDs, GCDs, YCDs, and RCDs.

To further demonstrate that WCDs are mixtures of different‐color‐emitting CDs, the WCDs were separated and purified by column chromatography, and details are shown in the Experimental Section. After separation, BCDs (430 nm), GCDs (507 nm), YCDs (540 nm), and RCDs (600 nm) were obtained. All of the CDs are well‐dispersed with average sizes of 17.12 nm (BCDs), 22.68 nm (GCDs), 4.87 nm (YCDs), and 2.88 nm (RCDs), as shown in **Figure** **2**a–d. The significant differences in particle size suggest different formation processes of multicolor CDs,^[^ ^18b^ ^]^ which is consistent with the wide particle size distribution observed in the TEM image of WCDs (Figure 1a). And only RCDs show well‐resolved lattice fringes with a lattice distance of 0.21 nm (inset in Figure 2d), corresponding to the (100) in‐plane lattice of graphite carbon,^[^ ^19^ ^]^ indicating that RCDs possess highly graphitized and crystallized carbon core. The high graphitization degree of RCDs means the increase of the conjugation degree and the content of sp^2^ hybrid carbon, resulting in a narrow bandgap (*E*_gap_) and long emission wavelength (*λ*_em_).^[^ ^20^ ^]^ Therefore, it is inferred that the fluorescence of RCDs mainly originates from the carbon core state.^[^ ^21^ ^]^ Based on the quantum confinement effect,^[^ ^22^ ^]^ if the PL mechanisms of BCDs, GCDs, and YCDs all belong to the carbon core state, their particle size should increase sequentially with the redshift *λ*_em_. Obviously, this is not in line with what we observed with TEM images. That is, the emission mechanisms of these CDs can't be unified by the carbon core state theory and may be related to the surface state.

To further figure out the emission mechanisms of all CDs, the structures of multicolor CDs were further characterized by X‐ray diffraction (XRD) patterns and Raman spectra. As shown in Figure 2e, BCDs, GCDs, and YCDs show a broad diffraction band centered at about 24.5°, corresponding to the (002) crystal plane of graphite structure demonstrating amorphous structures of CDs.^[^ ^23^ ^]^ RCDs show a relatively narrower half‐peak width at about 24.5° than the other three CDs, indicating that their carbon cores are tightly packed through sp^2^ conjugation structures, which means a higher degree of graphitization, corresponding to the HRTEM results.^[^ ^16b^ ^]^ The Raman spectra of multicolor CDs exhibit an obvious D band at 1262 cm^−1^ and a G band at 1607 cm^−1^ that corresponds to disordered structures and graphitic structures of carbon materials (Figure 2f).^[^ ^24^ ^]^ The ratio of *I*_G_/*I*_D_ is 1.01, 1.16, 1.38, and 1.27 for BCDs, GCDs, YCDs, and RCDs, respectively, indicating that the crystallinity of CDs is related to the redshift of wavelength.^[^ ^25^ ^]^ For these four CDs, although the size of CDs tends to increase and then decrease, the graphitization degree of CDs increases sequentially according to the appearance of lattice stripes in RCDs from HRTEM and XRD results. Especially for RCDs, the high degree of graphitization represents an increase in the effective conjugation domain, which makes their *λ*_em_ longer.^[^ ^26^ ^]^

The surface functional groups and chemical structures of multicolor CDs were investigated by Fourier transform infrared spectroscopy (FTIR) and X‐ray photoelectron spectroscopy (XPS) spectra. The FTIR spectra reveal that oxygen‐ and nitrogen‐containing functional groups are present on the surfaces of multicolor CDs. As shown in Figure 2g, all four CDs show C=O stretching vibrations of the amide I band at 1641 cm^−1^ and –NH bending vibrations of the amide II band at 1549 cm^−1^, demonstrating the formation of the amide bond and thus the occurrence of the dehydration condensation reaction during the formation process.^[^ ^27^ ^]^ From BCDs to YCDs, the enhanced intensity of amide I band and II band means an increase in the amide contents, and the intensity of C–O–C stretching vibrations at 1282 cm^−1^ relatively decreases, which are possibly responsible for the redshifted *λ*_em_.^[^ ^28^ ^]^ For BCDs and GCDs, a stretching vibration band at 1724 cm^−1^ from C=O and a bending vibration band at 1423 cm^−1^ from the –OH of carboxylic acid, showing that BCDs and GCDs are embellished with carboxyl groups. Meanwhile, YCDs show a broad and integrated stretching vibrational band at 3440 cm^−1^, implying the presence of a large number of hydrogen bonds.^[^ ^29^ ^]^ For RCDs, there are relatively weak amide, carboxyl, and C–O–C vibrational peaks, but much stronger stretching vibration bands of O–H and N–H at 3732 and 3184 cm^−1^.^[^ ^30^ ^]^ Meanwhile, a strong =C–H stretching vibration band in the aromatic ring appears at 2922 cm^−1^, which implies that RCDs have large conjugated structures, further indicating that the large conjugated structures cause the PL emission redshift of CDs.

**Figure 3:**
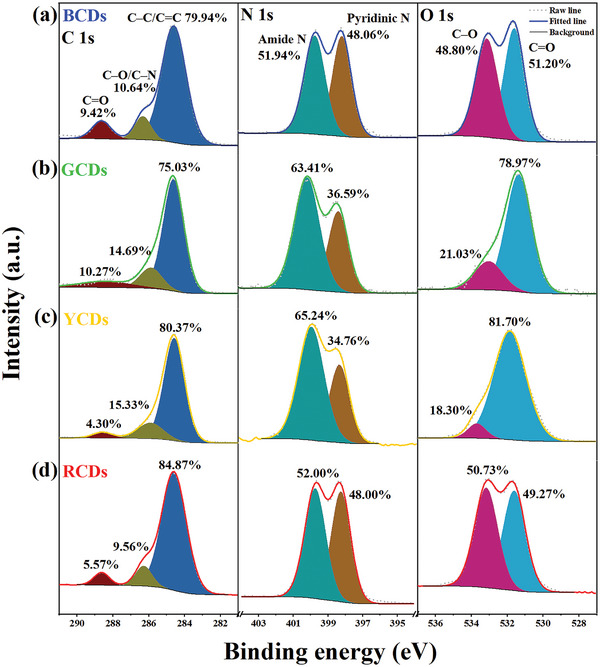
a–d) High‐resolution XPS C 1s, N 1s, and O 1s spectra of a) BCDs, b) GCDs, c) YCDs, and d) RCDs, respectively.

The full scan XPS of four CDs demonstrates the presence of carbon, nitrogen, and oxygen elements (Figure S2 and Table S1, Supporting Information). The high carbon content (> 73.5%) and low oxygen content (< 16.5%) further confirm that all four CDs undergo the carbonization process. The types of surface groups were analyzed by high‐resolution XPS spectra (**Figure** **3** and Tables S2–S4, Supporting Information). The C 1s spectra of multicolor CDs are all deconvoluted into three components, corresponding to C–C/C=C groups (284.6 eV), C−O/C–N groups (286.2 eV), and C=O groups (288.6 eV).^[^ ^31^ ^]^ The N 1s spectra are deconvoluted into two components at 398.3 and 399.7 eV, representing pyridinic N and amide N, respectively.^[^ ^29^ ^]^ The deconvolution of O 1s spectra can resolve two peaks centered at 531.6 and 533.3 eV corresponding to C=O and C–O.^[^ ^32^ ^]^ It is confirmed that all four CDs possess these functional groups on their surface, but have significantly different relative contents. The high‐resolution C1s spectra and O1s spectra show that BCDs and GCDs present more carboxylic acid groups than YCDs and RCDs. Besides, amide bonds exist in all four CDs (O=C–N, 286.2 eV in C1s, 399.7 eV in N1s, and 531.6 eV in O1s), and their content gradually increases from BCDs to YCDs, consistent with the FTIR results.

**Figure 4:**
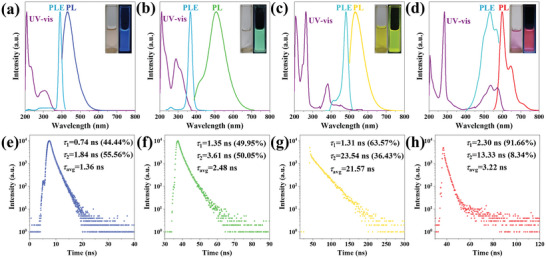
a–d) Normalized UV–vis absorption, PLE, and PL spectra of a) BCDs, b) GCDs, c) YCDs, and d) RCDs. Insets are the corresponding photographs of multicolor CDs in ethanol solution in daylight (left) and under a 400 nm lamp (right). e–h) TRPL curves of e) BCDs, f) GCDs, g) YCDs, and h) RCDs.

To make clear why these CDs show different emission colors, the photophysical properties were investigated by UV–vis absorption and steady/transient‐state fluorescence spectroscopy. **Figure** **4**a–d shows the normalized profiles of UV–vis, PL, and PL excitation (PLE) spectra of these four CDs. All of the CDs show a strong absorption band in the UVC (UVC: 200−280 nm) region, belonging to (*π*, π*) transitions of C=C/C=N double bonds owing to larger molar extinction coefficient.^[^ ^33^ ^]^ Besides, there exist relatively weak and distinct absorption bands in the long‐wavelength region. For BCDs and GCDs, the long‐wavelength absorption bands are located at about 300 nm with a low oscillator strength, which is attributed to the (*n*, π*) transitions of C=O.^[^ ^34^ ^]^ Moreover, the PLE peak is very close to this absorption band (Figure 4a,b), indicating that the PL mainly originates from the (*n*, π*) transitions of surface chromophores. It is demonstrated that the PL emission of BCDs and GCDs are mainly attributed to the surface state. For YCDs and RCDs, the long‐wavelength absorption bands appear in the blue (400–500 nm) and yellow (500–600 nm) region, and the distinct red‐shifted absorption bands are attributed to (*π*, π*) transitions of the large *π*‐conjugated sp^2^ aromatic system.^[^ ^35^ ^]^ For RCDs, the PLE peak match this (*π*, π*) transition mode very well, further confirming that the PL mechanism of RCDs belongs to the carbon core state. Based on this, the *E*_gap_ of BCDs, GCDs, YCDs, and RCDs calculated by UV–vis spectra are 3.50, 3.47, 2.17, and 2.06 eV (Figure S3, Supporting Information), respectively. The decreasing *E*_gap_ is consistent with their progressively redshifted PL spectra. The PL spectra of BCDs, GCDs, YCDs, and RCDs show obvious excitation‐independent emission peaks at 430, 507, 540, and 600 nm, respectively (Figure 4a–d and Figure S4, Supporting Information). Meanwhile, there is only a small overlap between the absorption and emission spectra, thus effectively avoiding self‐absorption and achieving strong luminescence emission.^[^ ^36^ ^]^ The emission peaks of multicolor CDs match well with those that appeared in the WCDs (Figure 1b), confirming that no other complexes are generated in WCDs.

Furthermore, the time‐resolved PL decay (TRPL) curves of BCDs, GCDs, YCDs, and RCDs were tested at the *λ*_ex_ of 375 nm. As shown in Figure 4e–h, the average fluorescence lifetimes of the four CDs are 1.36, 2.48, 21.57, and 3.22 ns, respectively, and the curves are fitted with similar double‐exponential functions. The detailed parameters are shown in Table S5, Supporting Information. Among the fluorescence decay lifetimes, the shorter decay lifetime (*τ*_1_) suggests that the fluorescence of CDs may originate from the radiative recombination of eigenstates associated with the carbon core, while the longer decay lifetime (*τ*_2_) is related to the recombination of surface state.^[^ ^18b^ ^]^ The percentage of *τ*_1_ gradually increases with the red shift of the PL spectra of CDs, indicating that the carbon core state gradually dominates the emission. In particular, *τ*_1_ of RCDs accounted for a relatively large proportion (91.66%), further indicating that the fluorescence mainly originates from the conjugated sp^2^ structure. For YCDs, as illustrated in the FTIR, there are abundant hydrogen bonds that can inhibit the rotation and vibration of their radiation centers, as well as nonradiative transitions of singlet excitons, stabilizing the excited singlet states, and further prolonging the lifetime.^[^ ^12^ ^]^

**Figure 5:**
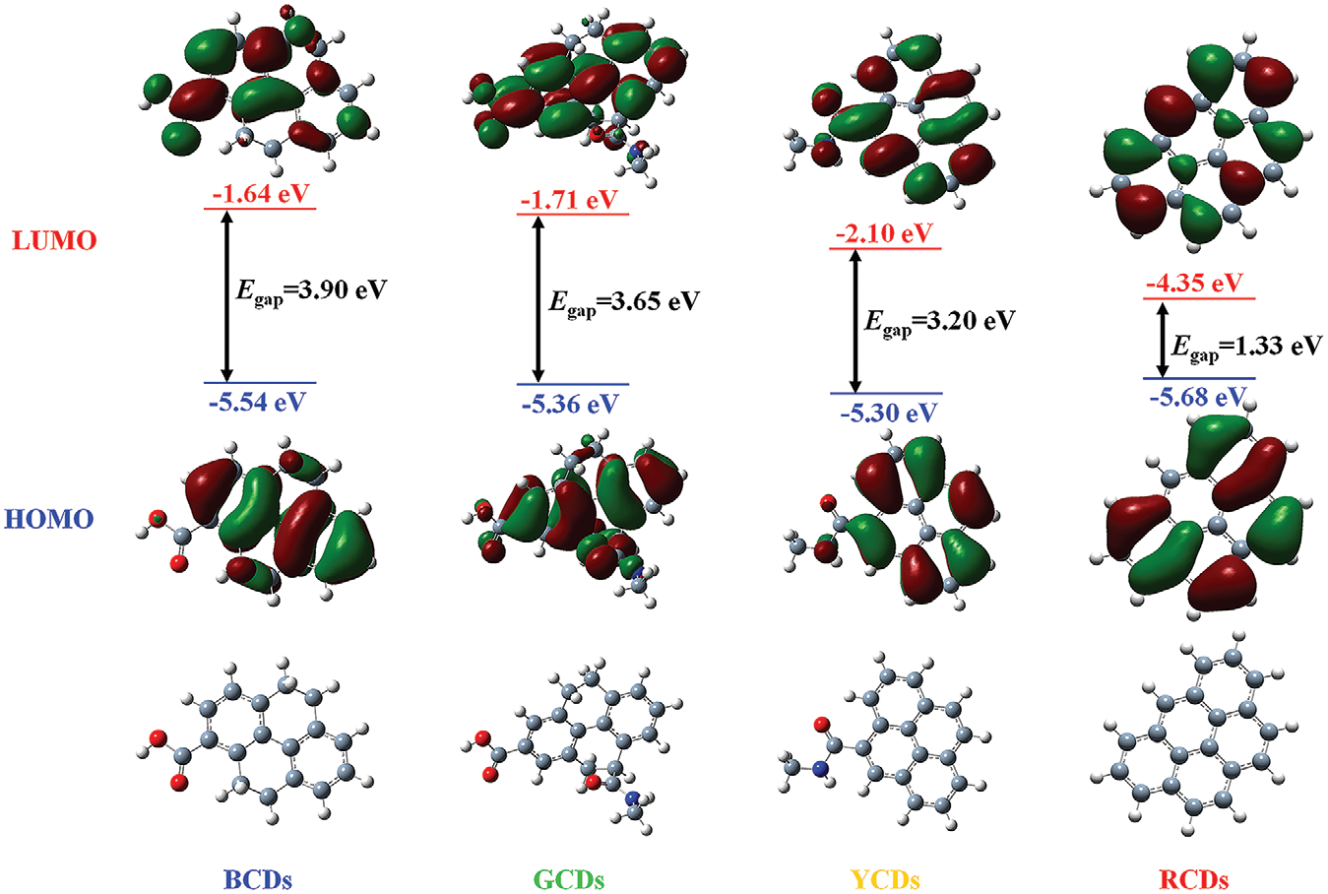
Theoretical calculations based on the pyrene model of BCDs, GCDs, YCDs, and RCDs at (TD‐DFT) B3LYP/6‐31G(d,p) level. HOMO: the highest occupied molecular orbital, LUMO: the lowest unoccupied molecular orbital.

To further elucidate the effect of the sp^2^ conjugation domain and amide structure on the *E*_gap_ and PL properties of CDs, the theoretical calculations were performed by using time‐dependent density functional theory (TD‐DFT) and the results were analyzed by Multiwfn.^[^ ^37^ ^]^ A typical model of pyrene‐based models,^[^ ^38^ ^]^ representing an sp^2^ domain with four fused aromatic rings that are further functionalized by different numbers of amide and carboxylic acid groups, was chosen to simulate the *E*_gap_ and emission wavelength of CDs (see Supporting Information for computational details). It is necessary to note that the simplified pyrene‐based model (about 1 nm) relative to CDs with larger sizes can be applied to reveal the relative trends, assisting in interpreting the PL mechanism of the CDs. Firstly, to illustrate the effect of a single variable, that is, the degree of conjugation or amide contents on *E*_gap_, the excited states and HOMO and LUMO energy levels of pyrene models with different sp^2^ domains and amide contents were calculated. As shown in Figure S5, Supporting Information, the *E*_gap_ decreases significantly with increasing the degree of conjugation or amide contents, which is consistent with the above analytical results. Secondly, the model structures of multicolor CDs were further designed based on the above experimental results, and the details were provided in the “Computational Details” section of Supporting Information. As shown in **Figure** **5** and Figure S6, Supporting Information, the calculated *E*_gap_ and *λ*_em_ are respective 3.90 eV/364 nm for BCDs, 3.65 eV/389 nm for GCDs, 3.20 eV/415 nm for YCDs, and 1.33 eV/612 nm for RCDs. It shows that the *E*_gap_ decreases and the *λ*_em_ redshifts with the increase of sp^2^ conjugation domain and amide contents, which is consistent with the experimental analysis and further proves the PL mechanism of CDs.

**Figure 6:**
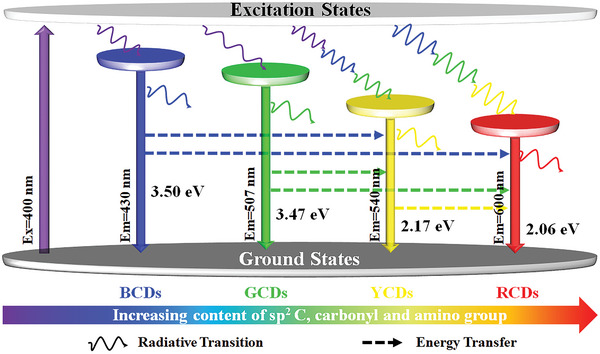
Schematic illustration of electron transition and energy transfer of multicolor CDs.

Taken together, the PL mechanisms of the four CDs can be explained by the synergistic interaction of carbon core and surface states. The surface state acts as a trapping center for excitons to produce surface defect state fluorescence, and more surface defects lead to a redshift of *λ*_em_. For BCDs and GCDs, despite the presence of a large number of carboxyl groups, the absence of conjugation effects and the low degree of carbonization result in a shorter *λ*_em_.^[^ ^39^ ^]^ From BCDs to YCDs, with the increase of surface amide contents, the degree of conjugation between the lone pair electrons of surface groups and the *π*‐conjugated electrons of carbon core increases, resulting in *E*_gap_ decreases, and thereby a redshift of *λ*_em_. Moreover, the strong hydrogen bonding interaction in YCDs rigidifies the molecular conformation, which facilitates the redshift of the emission peak. For RCDs with a large sp^2^ conjugation domain, an increase in the degree of *π*‐electron delocalization reduces the *E*_gap_, leading to red light emission. Returning to WCDs, it has been confirmed that the emission components of WCDs originate from multicolor CDs, and the PL emission peaks of WCDs are the superposition of multicolor CDs. But the PLE spectra of multicolor CDs show that their optimal excitation wavelengths are not overlapping. Therefore, for WCDs, the white light emission at the *λ*_ex_ of 400 nm may be the result of other interactions or energy transfers between multicolor CDs. It is generally accepted that FRET leads to the redshift, which depends largely on the spectral overlap between the absorption band of the acceptor and the emission band of the donor.^[^ ^40^ ^]^ When *λ*_ex_ is 400 nm, the electrons of BCDs and GCDs can be efficiently excited to emit fluorescence at 430 and 507 nm, which overlap well with the optimal *λ*_ex_ of YCDs and RCDs (Figure S7, Supporting Information). The FRET between CDs eventually leads to the PL reduction of donor CDs (BCDs, GCDs) and PL enhancement of acceptor CDs (YCDs, RCDs), which is the reason that YCDs and RCDs can still achieve PL emission and intensity enhancement in the presence of a large stokes shift (> 200 nm). The FRET process eventually helps to balance the PL intensity of multicolor CDs and achieve white light emission (**Figure** **6**). The FRET process can be confirmed by the normalized PL spectra of WCDs at different concentrations (Figure S8, Supporting Information). As the concentration increases, relative to the normalized short‐wavelength emission peak (450 nm), the intensities of the long‐wavelength emission peak (599 nm) enhance obviously, confirming the existence of the process of FRET.

**Figure 7:**
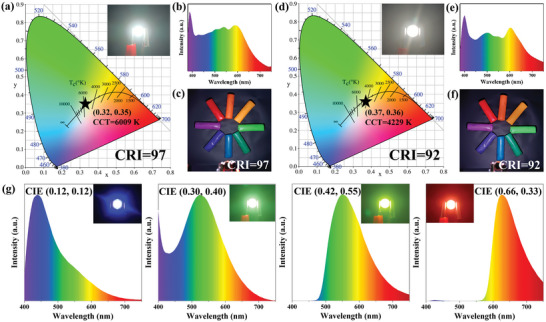
CIE coordinates of LEDs prepared using a) WCDs and d) mixtures of multicolor CDs as phosphors. Insets are the corresponding digital photographs of WLEDs. b) and e) are the corresponding electroluminescence spectra. c) and f) are the corresponding digital photographs of colored plasticine. g) Electroluminescence spectra of LEDs prepared using BCDs, GCDs, YCDs, and RCDs as phosphors. Insets are the corresponding digital photographs of multicolor LEDs.

The intense white light emission and facile purification process of WCDs are notable for their use in high‐performance WLEDs. A 380 nm LED chip was selected as the excitation source and combined with a mixture of WCDs and polyvinylpyrrolidone (PVP) to fabricate WLEDs, the detailed fabrication process was provided in the Experimental Section. As shown in **Figure** **7**a, the WLEDs exhibit excellent white light illumination performances with CIE coordinates of (0.32, 0.35) and CCT of 6009 K. In particular, the CRI of WLEDs is as high as 97, which is almost the highest value among reported WLEDs in recent years, especially in the field of WCDs‐based WLEDs (Table S6, Supporting Information). The high CRI is ascribed to broad visible light emission (Figure 7b), and the emission spectra cover the entire visible region from 390 to 750 nm, which is comparable to natural light. The emission spectra of WLEDs show four major emission peaks at 430, 505, 540, and 596 nm except for the peak of the 380 nm LED chip, which is close to the PL spectra of the WCDs solution. This indicates that the multicolor CDs components in WCDs retain constant fluorescence properties in the PVP matrix. When the WLEDs are used to illuminate colored plasticine under dark conditions (Figure 7c), the colors are clearly distinguishable and show the true colors of the objects well, reflecting the high color rendering quality of the WLEDs. Furthermore, the performance parameters of WLEDs at different working voltages were measured to investigate the effect of energy transfer on the color stability of WLEDs (Table S7, Supporting Information). As shown in Figure S9a, Supporting Information, the electroluminescence intensities of WLEDs increase with increasing forward voltage from 3.20 to 3.50 V. The maximum brightness of luminescence is 50 110 cd m^−2^ (Figure S9b, Supporting Information), which is much larger than that of the WLEDs reported so far.^[^ ^41^ ^]^ With the increase of voltage, the CIE coordinates of the WLEDs only shift slightly from (0.33, 0.35) to (0.30, 0.32) (Figure S9c, Supporting Information), and the CRI only changes slightly between 96 and 97 (Figure S9b, Supporting Information), showing the excellent color stability of the WLEDs. Meanwhile, the thermal stability of WCDs (Figure S10, Supporting Information) was tested, and the results showed that when the temperature continued to rise to 200 °C, the weight of WCDs could still retain 97.87%, demonstrating excellent thermal stability.

To further confirm that the isolated multicolor CDs can also be used to fabricate WLEDs with high CRI after remixing again, BCDs, GCDs, YCDs, and RCDs were mixed in accordance with a certain mass ratio of 3:5:2:3 and re‐prepared as phosphors for WLED. As shown in Figure 7d,e, the WLED with CIE coordinates of (0.37, 0.36), CCT of 4229 K, and CRI of 92 were obtained. Compared to the WLED constructed directly using WCDs as phosphors, the values of CRI and CCT decrease slightly owing to the difficulty in tuning the ratio. But the PL peak positions remain constant (Figure 7b,e) and the true colors of plasticine display very well (Figure 7f), confirming that the WLEDs can be achieved again by this method. Compared with this method, the WLED from direct WCDs is free from going through a complicated ratio regulation process, which significantly saves time and cost. In addition, the multicolor CDs can also be used to construct multicolor LEDs with CIE coordinates of (0.12, 012), (0.30, 0.40), (0.42, 0.55), and (0.66, 0.33), respectively (Figure 7g), which greatly broadens the application prospects of multicolor CDs and WCDs. These results provide strong evidence of the excellent potential of CDs‐based phosphors in high‐performance LEDs.

## 3 Conclusion

In summary, a facile one‐step synthesis method was developed to prepare WCDs covering the entire visible light, which is composed of BCDs, GCDs, YCDs, and RCDs. The FRET process between multicolor CDs constructs a balance emission and induces the generation of white light. The theoretical calculations and experiments confirm that the PL emission of multicolor CDs is attributed to the synergistic effect of the carbon core and surface states. For surface state, amide contents play a key role in promoting the redshift of *λ*_em_. The carbon core state dominates the emission of RCDs, while the emission wavelength redshifts with increasing graphitization. Subsequently, the application of WCDs is demonstrated for constructing WLEDs, which exhibit excellent white‐light illumination performance with CIE coordinates of (0.32, 0.35), CCT of 6009 K, and CRI as high as 97. A series of multicolor LEDs were also obtained by using the multicolor CDs as single phosphors. This work will provide an advanced and highly effective way for the direct application of WCDs in high‐performance WLEDs.

## 4 Experimental Section

### Chemicals and Materials

TMLA and *o*‐PD were obtained from Shanghai Macleans Biochemical Technology Co., Ltd. (Shanghai, P. R. China). Ethanol and ethyl acetate were obtained from Tianjin Tianli Chemical Reagent Co., Ltd. (Tianjin, P. R. China). PVP was purchased from Sigma Aldrich (Shanghai) Trading Co., Ltd. (Shanghai, P. R. China). All reagents were of analytical grade and used directly without further purification. Deionized water was used for all experiments.

### Characterization

TEM and HRTEM images were obtained with a JEOL JEM‐2010 transmission electron microscope. XRD patterns were carried out by X‐ray diffraction using Cu K*α* radiation (Rigaku‐D/Max 2500). Raman spectra were recorded using a HORIB A Evolution Raman spectrometer. FTIR spectra were collected by a TENSOR2 spectrometer. XPS curves were obtained on an AXIS ULTRA DLD X‐ray diffractometer. UV–vis spectra were recorded using a HITACHI U‐3900 spectrophotometer. The PL spectra were collected on the HORIBA FluoroMax‐4 fluorescence spectrophotometer. The TRPL curves were measured on an EDINBURGH FLS980 spectrometer. The photoelectric properties of the LEDs were measured by Spectrascan PR655. The thermogravimetric curve was measured using a Setaram Labsys Evo thermogravimetric analyzer with a heating rate of 10 °C min^−1^ in a nitrogen atmosphere.

### Preparation of WCDs

WCDs were synthesized by a one‐step solvothermal method. TMLA (0.21 g, 1.00 mmol) and *o*‐PD (0.22 g, 2.00 mmol) were dissolved in 20 mL ethanol and ultrasound for 5 min. Then the mixture was transferred into a 50 mL Teflon‐lined autoclave and heated at 200 °C for 10 h. After cooling to room temperature, the products were filtered through a 0.22 µm microporous membrane to obtain the WCDs solution. The hydrophobicity of WCDs can be used to obtain WCDs solid powder quickly. Finally, the solid WCDs powder was precipitated in excess deionized water, filtered, and dried at 40 °C under vacuum to a constant weight.

### Preparation of Multicolor CDs

Multicolor CDs were prepared by the column chromatography of WCDs. Thin‐layer chromatography was used to verify the components of WCDs and to determine the type and proportion of eluents. Then, the WCDs were eluted with a gradient of ethyl acetate and ethanol as elutes. The solid multicolor CDs were prepared by vacuum distillation to remove ethyl acetate and ethanol and dried at 40 °C under vacuum to a constant weight. Multicolor CDs were named BCDs, RCDs, GCDs, and YCDs according to their emission colors.

### Preparation of CDs/PVP Complex Solution

The WCDs/PVP complex solution was prepared as follows: WCDs (0.30 mg) were mixed in PVP ethanol solution (0.25 g mL^−1^, 120 µL) under ultrasonic treatment for 5 min to obtain WCDs/PVP mixed solution. The WCDs/PVP complex solution was prepared from isolated multicolor CDs as follows: BCDs (0.30 mg), GCDs (0.50 mg), YCDs (0.20 mg), and RCDs (0.30 mg) were mixed in PVP ethanol solution (0.25 g mL^−1^, 120 µL) under ultrasonic treatment for 5 min to obtain WCDs/PVP complex solution. The multicolor CDs/PVP complex solution was prepared from a mixture of monochrome CDs and PVP ethanol solution under ultrasonic for 5 min.

### Fabrication of WLEDs and Multicolor LEDs

A total of six types of LEDs were prepared, including two types of WLEDs and four types of monochrome LEDs fabricated with the same processes. Specifically, WCDs/PVP mixed solution (40 µL) was dropped into the LED lamp cup, and after natural drying, continued to drop and dry until 120 µL WCDs/PVP complex solution was dropped into the LED lamp cup. After completely drying, the lamp cup was installed on the LED chip (380 nm), and assembled into WLEDs. The fabrication method of multicolor LEDs is the same as that of WLEDs.

## Conflict of Interest

The authors declare no conflict of interest.

## Supporting information

Supporting information

## Data Availability

The data that support the findings of this study are available in the supplementary material of this article.
