# Supplementary material for: One‐Step Synthesis of White‐Light‐Emitting Carbon Dots for White LEDs with a High Color Rendering Index of 97
Source: Adv Sci (Weinh). 2023 Feb 23;10(12):2206386. doi: 10.1002/advs.202206386 (PMC10131834; doi:10.1002/advs.202206386)
Supplement: Supplementary file 1 — Supporting information [file ADVS-10-2206386-s001.pdf]

ADVANCED SCIENCE
Open Access

Supporting Information

for *Adv. Sci.*, DOI 10.1002/advs.202206386

One-Step Synthesis of White-Light-Emitting Carbon Dots for White LEDs with a High Color Rendering Index of 97

Zishan Yan, Tong Chen, Lingpeng Yan, Xinghua Liu, Jingxia Zheng, Fu-de Ren, Yongzhen Yang, Bin Liu*, Xuguang Liu and Bingshe Xu*

Supporting Information

One-Step Synthesis of White-Light-Emitting Carbon Dots for White LEDs with a High Color Rendering Index of 97

Zishan Yan, Tong Chen, Lingpeng Yan, Xinghua Liu, Jingxia Zheng, Fu-de Ren, Yongzhen Yang, Bin Liu*, Xuguang Liu, and Bingshe Xu*

Computational Details

All the calculations were performed with the time-dependent density functional theory (TD-DFT) method at B3LYP/6-31G(d,p) level using Gaussian 09 program. Multiwfn software is applied to analysis the emission wavelength.[1]

The choice of the calculation model is based on the following principles: a typical model of pyrene-based models,[2] representing a sp^2 domain with four fused aromatic rings that are further functionalized by different numbers of amide and carboxylic acid groups, was chosen to simulate the bandgap (E_{gap}) and wavelength of carbon dots (CDs). First, to illustrate the effect of a single variable, two factors (i.e., degree of conjugation, amide contents) were considered. (1) The energy structures of pyrene models with different sp^2 domains were calculated, and three models containing 2, 4, and 5 aromatic rings were illustrated in Figure S5. (2) The energy structures of pyrene models with different amide contents (zero, one and two amide groups) were calculated and the corresponding structures of these models were also given in Figure S5.

Furthermore, according to the TEM images, XRD patterns and Raman spectra, the degree of conjugation of CDs gradually increases with the redshift of emission wavelength. Therefore, pyrene with larger π-conjugated structure was selected as the carbon core model of YCDs, 1,2,3,6,7,8-hexahydropyrene with smaller π-conjugated structure was selected as the carbon core model of BCDs and GCDs, and benzopyrene with the largest π-conjugated structure was selected as the carbon core model of RCDs. The surface groups of CDs were determined by the characterization results of FTIR and XPS spectra. The amide contents of B-/G-/YCDs increase sequentially and the surface of BCDs and GCDs contains carboxyl groups. One carboxyl group was attached on the 1,2,3,6,7,8-hexahydropyrene (i.e., the model of BCDs), one carboxyl group and one amide group were attached on the 1,2,3,6,7,8-hexahydropyrene

(i.e., the model of GCDs), and one amide group was attached on the pyrene (i.e., the model of YCDs). The corresponding structures of these models are given in Figure 5.

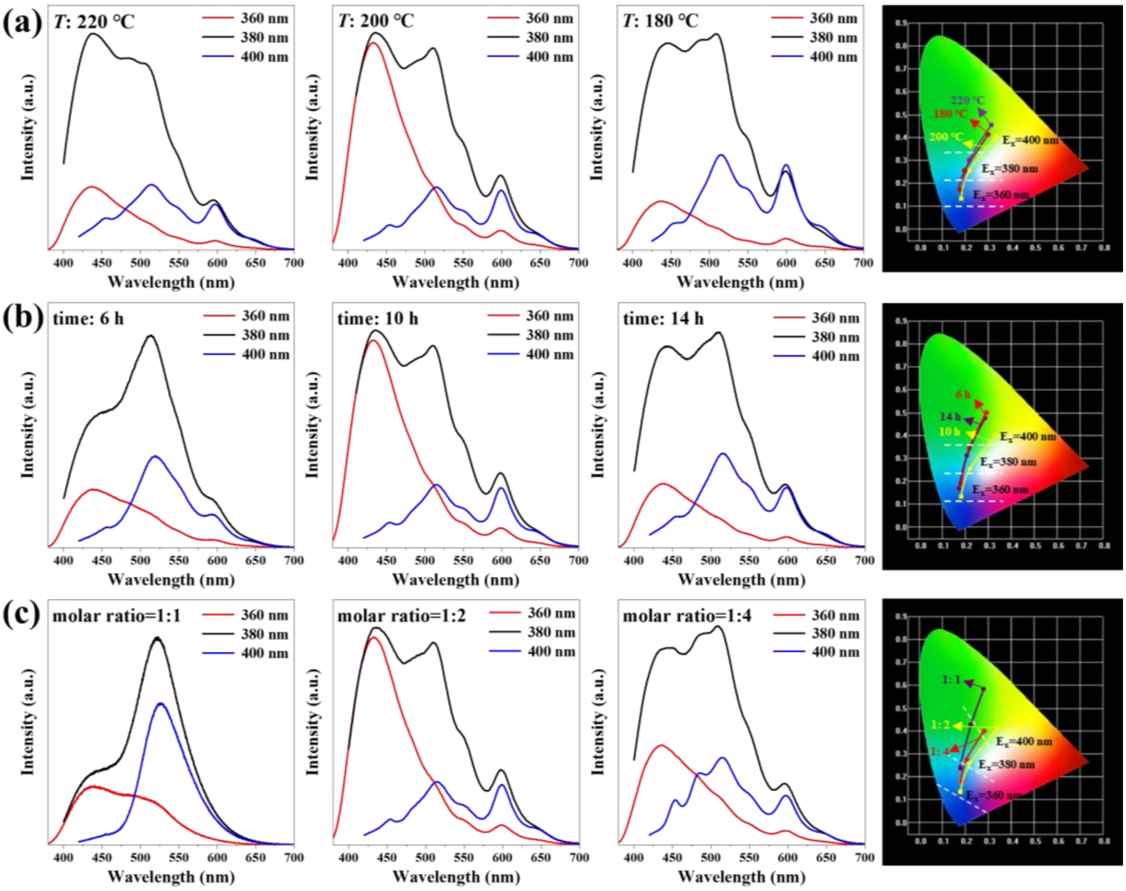

Figure S1. Photoluminescent (PL) spectra and Commission International de l'Eclairage (CIE) coordinates of sample solution (concentration: 0.375 mg mL^{-1}) under different (a) reaction temperature (*T*), (b) reaction time, and (c) molar ratio of trimellitic acid and *o*-phenylenediamine.

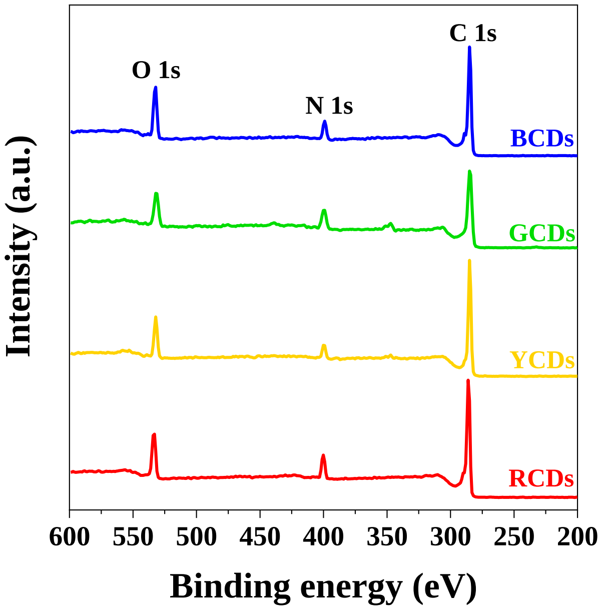

Figure S2. The full scanned XPS spectra of the four CDs.

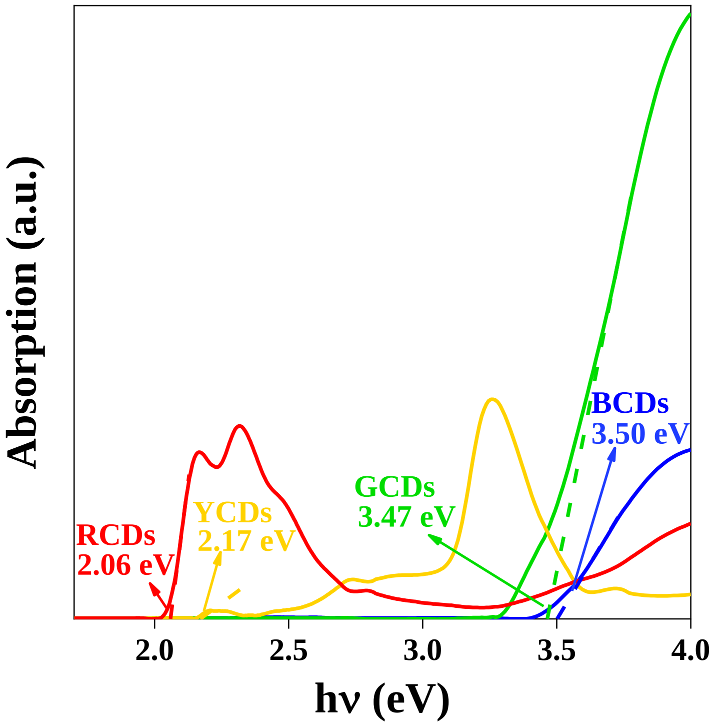

Figure S3. Determination of E_{gap} of the four CDs from UV-vis spectra. The E_{gap} of the four CDs were calculated using the equation $E_{gap}=1240/\lambda_{edge}$, where λ_{edge} is the wavelength of the maximum absorption edge.[3]

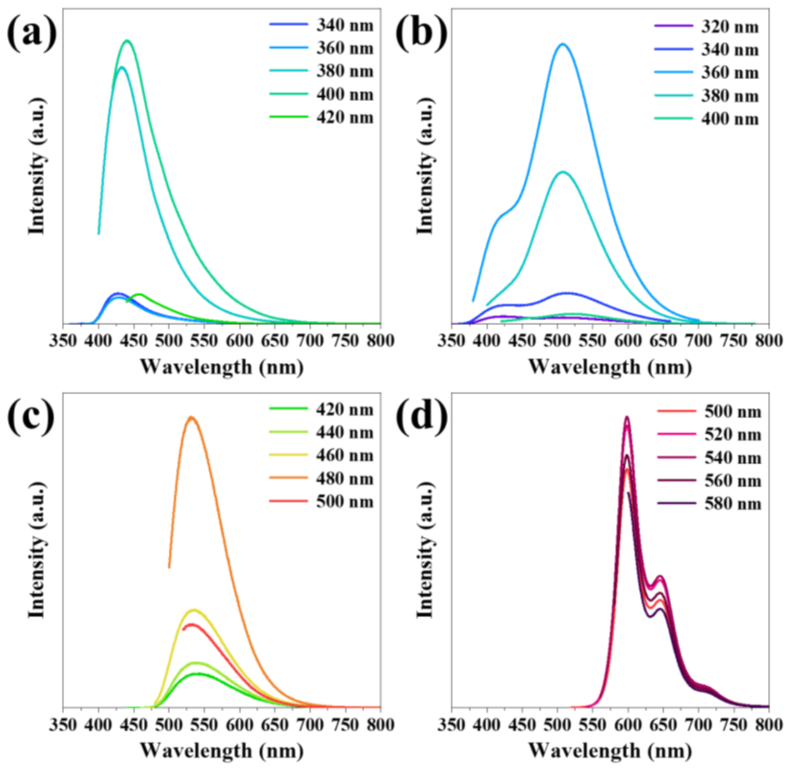

Figure S4. PL spectra of (a) BCDs, (b) GCDs, (c) YCDs, and (d) RCDs at different excitation wavelengths (λ_{ex}).

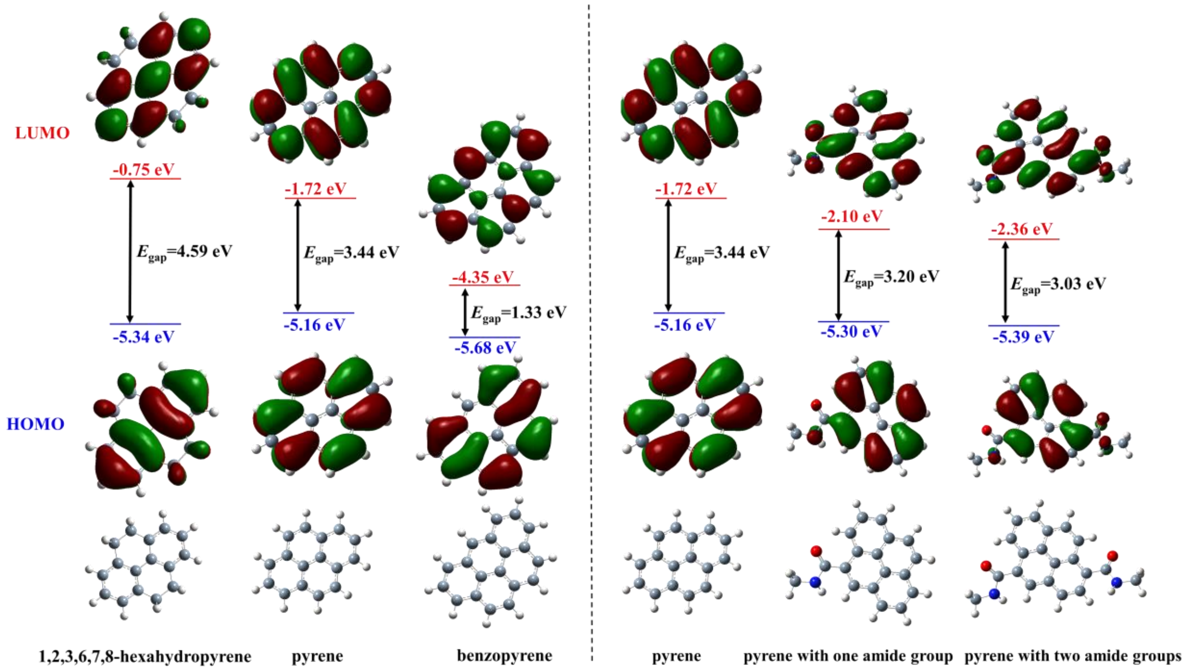

Figure S5. TD-DFT calculation results and the calculated HOMO and LUMO. HOMO: the highest occupied molecular orbital, LUMO: the lowest unoccupied molecular orbital.

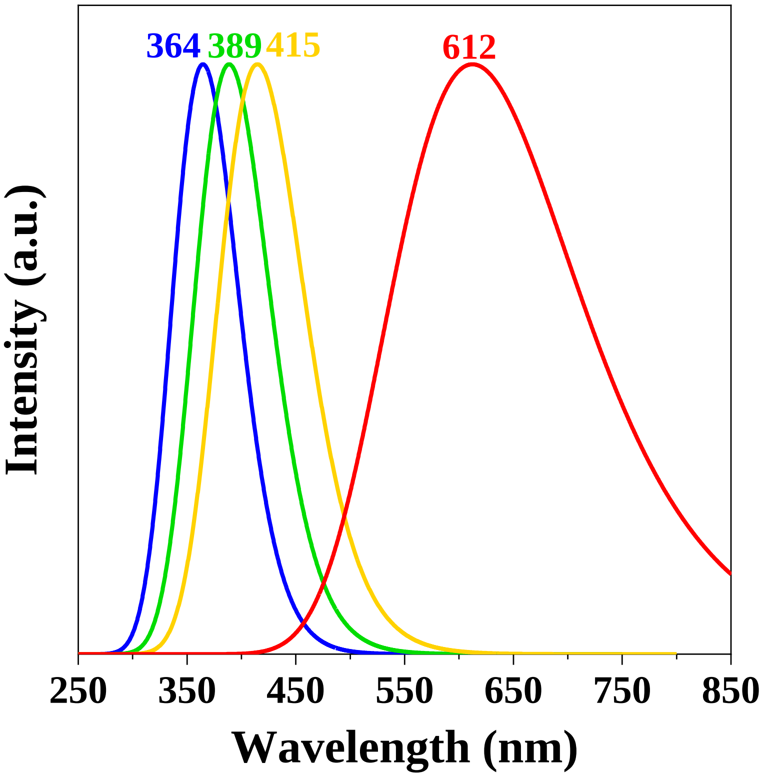

Figure S6. Normalized PL spectra of theoretical calculations based on pyrene model of BCDs, GCDs, YCDs and RCDs at (TD-DFT) B3LYP/6-31G(d,p) level.

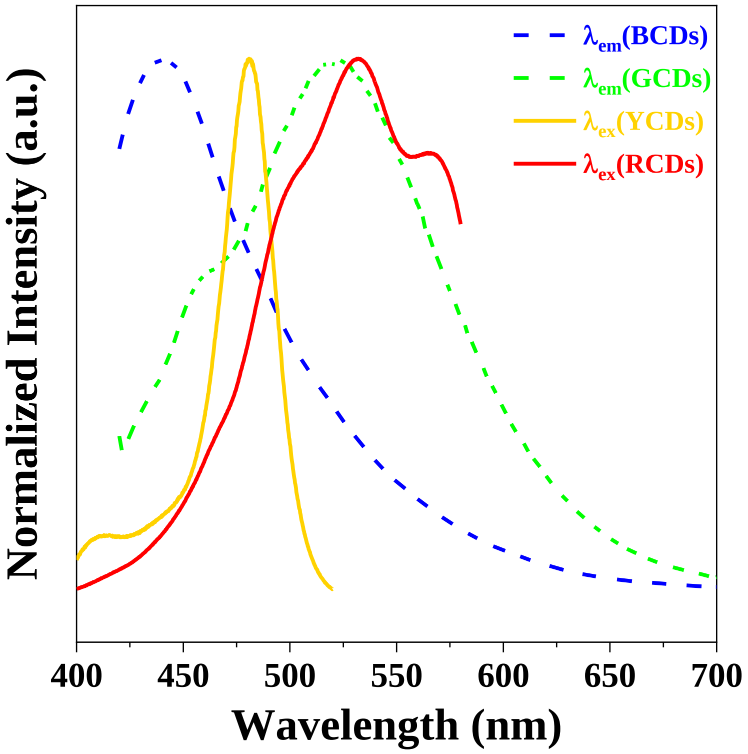

Figure S7. Overlap between PL spectra (BCDs and GCDs) and PL excitation spectra (YCDs and RCDs).

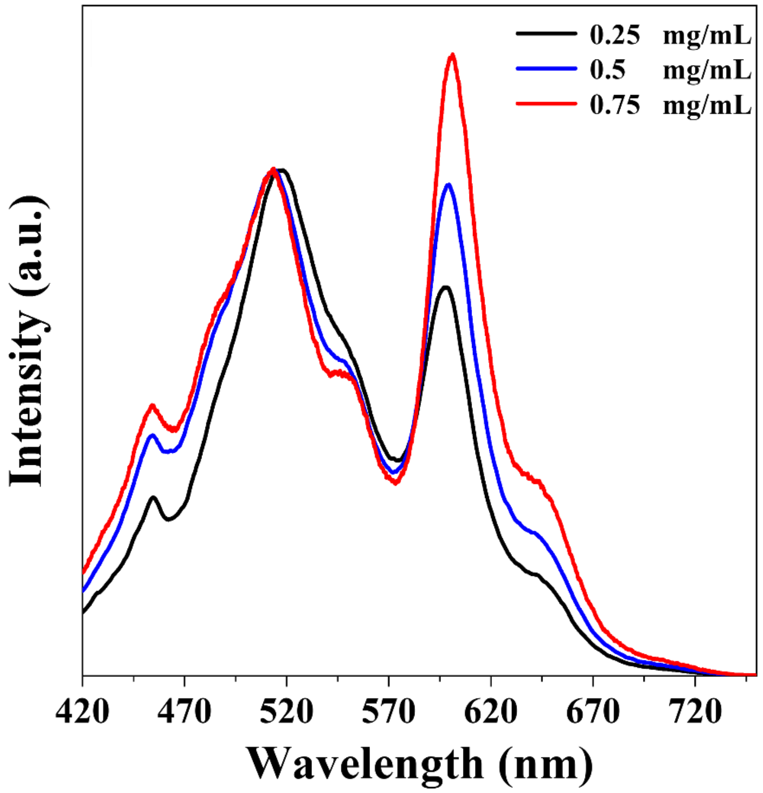

Figure S8. Normalized PL spectra of WCDs solutions with different concentrations (λ_{ex}=400 nm).

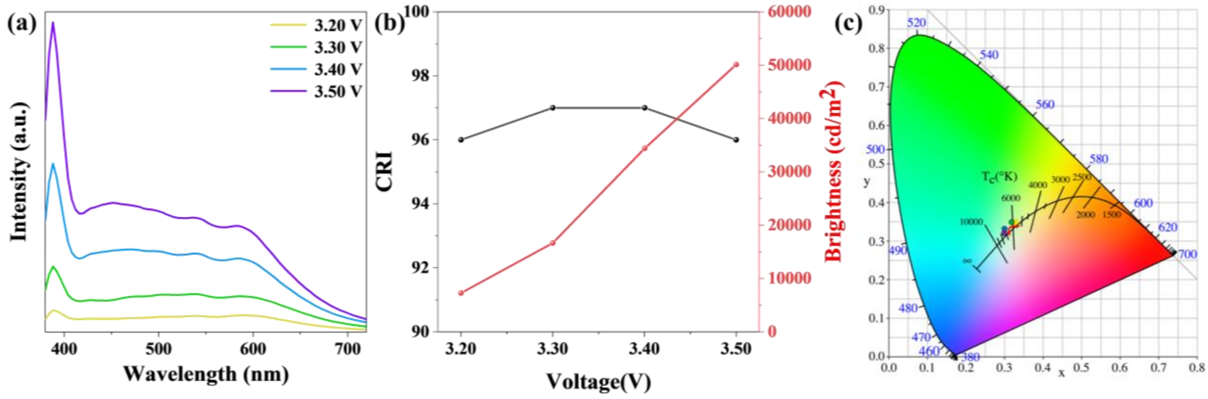

Figure S9. (a) Electroluminescence spectra, (b) brightness and CRI values and (c) CIE coordinates of WLEDs under different drive voltages from 3.20 to 3.50 V.

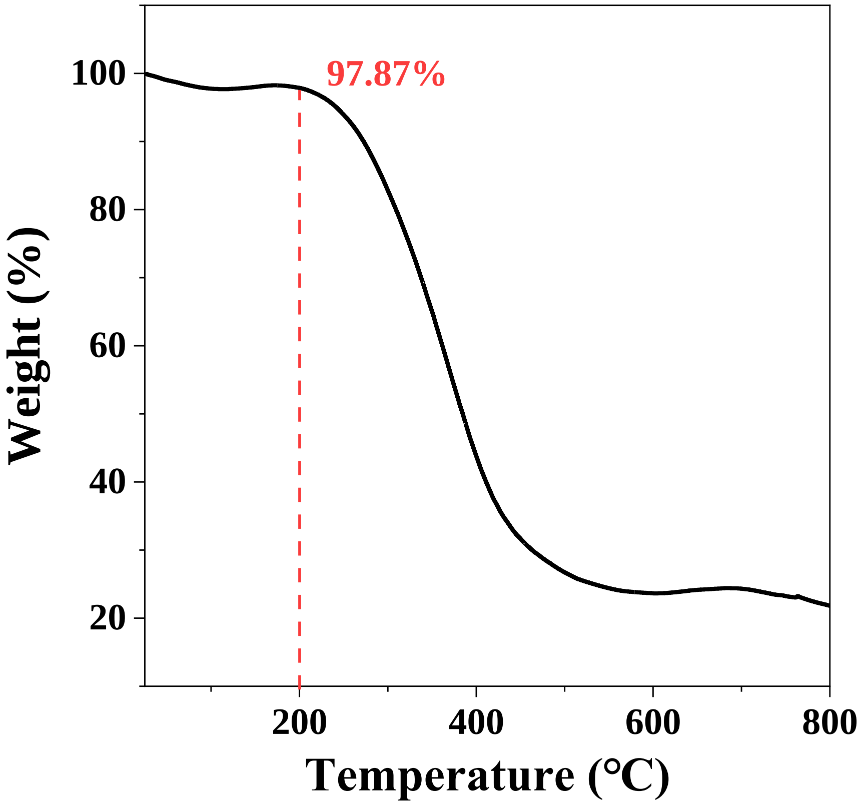

Figure S10. Thermogravimetry curve of WCDs. (heating rate of 10 °C min^{-1}, nitrogen atmosphere)

Table S1. Atomic relative content of the four CDs calculated by XPS spectra.

Sample	C (atomic %)	N (atomic %)	O (atomic %)
BCDs	74.63	8.89	16.47
GCDs	73.52	12.93	13.55
YCDs	78.87	7.80	7.80
RCDs	76.04	10.65	10.65

Table S2. XPS data analyses of the C 1s spectra of the four CDs.

Sample	C–C/C=C	C–O/C–N	C=O

BCDs	79.94%	10.64%	9.42%
GCDs	75.03%	14.69%	10.27%
YCDs	80.37%	15.33%	4.30%
RCDs	84.87%	9.56%	5.57%

Table S3. XPS data analyses of the N 1s spectra of the four CDs.

Sample	Pyridinic N	Amide N
BCDs	48.06%	51.94%
GCDs	36.59%	63.41%
YCDs	34.76%	65.24%
RCDs	48.00%	52.00%

Table S4. XPS data analyses of the O 1s spectra of the four CDs.

Sample	C=O	C–O
BCDs	51.20%	48.80%
GCDs	78.97%	21.03%
YCDs	81.70%	18.30%
RCDs	49.27%	50.73%

Table S5. Fluorescence lifetimes of the four CDs.

Sample	λ_{em} (nm)	τ_1 (ns)	B_1 (%)	τ_2 (ns)	B_2 (%)	χ^2	τ_{avg} (ns)
BCDs	430	0.74	44.44	1.85	55.56	1.143	1.36
GCDs	507	1.35	49.95	3.61	50.05	1.075	2.48
YCDs	540	1.31	63.57	23.54	36.43	1.097	21.57
RCDs	600	2.30	91.66	13.33	8.34	1.139	3.22

λ_{em}: emission wavelength, τ_1: the shorter decay lifetime, τ_2: the longer decay lifetime, τ_{avg}: the average decay lifetime, B_1 and B_2 are the fractional contributions of time resolved decay lifetime of τ_1 and τ_2.

The fluorescence lifetime of CDs was evaluated by a multidimensional time-correlated single photon counting approach, and then the test results were fitted using the following equation:

$$I(\mathrm{t})=\mathrm{B}_1 e^{(-\mathrm{t}/\tau_1)} + \mathrm{B}_2 e^{(-\mathrm{t}/\tau_2)} \qquad \text{(S1)}$$

Here I(t) is the PL intensity at time t. The value of the average lifetime τ_{avg} can be calculated by the following equation:

$$\tau_{avg}=\frac{\mathrm{B}_1\tau_1^2+\mathrm{B}_2\tau_2^2}{\mathrm{B}_1\tau_1+\mathrm{B}_2\tau_2} \qquad \text{(S2)}$$

Table S6. Summary of the performance parameters of WLEDs using white-light-emitting CDs as phosphors.

Precursor of white-light-emitting CDs	Pump source/nm	CCT/K	CRI	CIE	Ref.
poly(styrene-co-glycidylmethacrylat)	370	-	-	(0.34, 0.37)	4
poly(vinyl alcohol) and ethylenediamine	375	-	-	-	5
urea and citric acid	365	-	-	(0.31, 0.33)	6
citric acid and 1-(2-pyridylazo)-2-naphthol	365	-	-	(0.29, 0.31)	7
1,6-dihydroxynaphthalene and L-asparagine	365	-	-	(0.32, 0.31)	8
ammonium citrate and ethylenediamine tetraacetic acid	460	6565	68.4	(0.32, 0.33)	9
1,5-diaminonaphthalene and trichloromethane	390	3938	70.6	(0.39, 0.38)	10
lycorine hydrochloride and boric acid	370	5028	82.0	(0.34, 0.38)	11
urea and phosphoric acid	370	8756	85.3	(0.268, 0.346)	12
o-phenylenediamine and tartaric acid	365	5994	86.7	(0.33, 0.33)	13
ethanol	365	-	87.8	(0.37, 0.39)	14
guanidine carbonate and monopotassium phosphate	400	3032	91	(0.42, 0.38)	15
2,2′-dithiodibenzoic acid	390	-	89	(0.33, 0.30)	16
trimellitic acid and *o*-phenylenediamine	380	6009	**97**	(0.32, 0.35)	**This work**

Table S7. Performance parameters of WLEDs under different drive voltages from 3.20 to 3.50 V.

Voltage (V)	CIE (x, y)	CRI	CCT (K)	Brightness (cd m^{-2})	Luminous efficiency (lm W^{-1})
3.20	(0.33, 0.35)	96	5451	7279	4.24
3.30	(0.32, 0.35)	97	6009	16640	3.50
3.40	(0.30, 0.33)	97	6995	34410	3.83
3.50	(0.30, 0.32)	96	7643	50110	3.56

References

[1] Lu T, Chen F. Multiwfn: a multifunctional wavefunction analyzer[J]. Journal of computational chemistry, 2012, 33(5): 580–592.
[2] Hola K, Sudolská M, Kalytchuk S, et al. Graphitic nitrogen triggers red fluorescence in carbon dots[J]. ACS nano, 2017, 11(12): 12402–12410.
[3] Ding Y, Zheng J, Wang J, et al. Direct blending of multicolor carbon quantum dots into fluorescent films for white light emitting diodes with an adjustable correlated color temperature[J]. Journal of Materials Chemistry C, 2019, 7(6): 1502–1509.
[4] Guo X, Wang C F, Yu Z Y, et al. Facile access to versatile fluorescent carbon dots toward light-emitting diodes[J]. Chemical Communications, 2012, 48(21): 2692–2694.
[5] Chen Y, Zheng M, Xiao Y, et al. A self-quenching-resistant carbon-dot powder with tunable solid-state fluorescence and construction of dual-fluorescence morphologies for white light-emission[J]. Advanced Materials, 2016, 28(2): 312–318.
[6] Liu Y, Zhang T, Wang R, et al. A facile and universal strategy for preparation of long wavelength emission carbon dots[J]. Dalton Transactions, 2017, 46(48): 16905–16910.
[7] Yan F, Jiang Y, Sun X, et al. Multicolor carbon dots with concentration-tunable fluorescence and solvent-affected aggregation states for white light-emitting diodes[J]. Nano Research, 2020, 13(1): 52–60.
[8] Han B, Jiang J, Yan Q, et al. One-step straightfoward solid synthesis of high yield white fluorescent carbon dots for white light emitting diodes[J]. Chinese Chemical Letters, 2021, 32(2): 591–593.
[9] Zhang Y, Yuan R, He M, et al. Multicolour nitrogen-doped carbon dots: tunable photoluminescence and sandwich fluorescent glass-based light-emitting diodes[J]. Nanoscale, 2017, 9(45): 17849–17858.
[10] Li W, Guo H, Li G, et al. White luminescent single-crystalline chlorinated graphene quantum dots[J]. Nanoscale Horizons, 2020, 5(6): 928–933.
[11] Li Q, Li Y, Meng S, et al. Achieving 46% efficient white-light emissive carbon dot-based materials by enhancing phosphorescence for single-component white-light-emitting diodes[J]. Journal of Materials Chemistry C, 2021, 9(21): 6796–6801.
[12] Wang Z, Liu Y, Zhen S, et al. Gram-scale synthesis of 41% efficient single-component white-light-emissive carbonized polymer dots with hybrid fluorescence/phosphorescence for white light-emitting diodes[J]. Advanced Science, 2020, 7(4): 1902688.
[13] Wang L, Li W, Yin L, et al. Full-color fluorescent carbon quantum dots[J]. Science advances, 2020, 6(40): eabb6772.
[14] Wang B, Song H, Tang Z, et al. Ethanol-derived white emissive carbon dots: the formation process investigation and multi-color/white LEDs preparation[J]. Nano Research, 2022, 15(2): 942–949.
[15] Meng T, Yuan T, Li X, et al. Ultrabroad-band, red sufficient, solid white emission from carbon quantum dot aggregation for single component warm white light emitting diodes with a 91 high color rendering index[J]. Chemical Communications, 2019, 55(46): 6531–6534.
[16] Liu F, Xu S, Xia P, et al. Anhydride-terminated solid-state carbon dots with bright orange emission induced by weak excitonic electronic coupling[J]. ACS Applied Materials & Interfaces, 2022, 14(4): 5762–5774.
